# Multiple chronic diseases and psychological distress among adults in the United States: the intersectionality of chronic diseases, race/ethnicity, immigration, sex, and insurance coverage

**DOI:** 10.1007/s00127-024-02730-1

**Published:** 2024-07-17

**Authors:** David Adzrago, David R. Williams, Faustine Williams

**Affiliations:** 1 Division of Intramural Research, National Institute on Minority Health and Health Disparities, National Institutes of Health, Bethesda, MD USA; 2 Department of Social and Behavioral Sciences, Department of African and African American Studies, Harvard T.H. Chan School of Public Health, Harvard University, Cambridge, MA USA

**Keywords:** Mental health disparities, Immigration status, Chronic diseases, Intersectionality, Anxiety/depression, Physical health disparities

## Abstract

**Purpose:**

Psychological distress significantly contributes to the burdens of morbidity and mortality in the United States (U.S.), but our understanding is limited with regards to the risk factors associated with psychological distress. We used nationally representative data to examine (1) the comorbidities of chronic diseases and their risks for psychological distress and (2) the ways in which chronic diseases combine with demographic factors such as sex, race/ethnicity, immigration status, and health insurance coverage to affect the patterning of psychological distress.

**Methods:**

We analyzed the 2005–2018 National Health Survey Interview cross-sectional data on U.S. adults aged *≥* 18 years (*n* = 351,457). We fitted sequential multivariable logistic regression models.

**Results:**

There was a dose-response relationship between the number of chronic diseases and psychological distress, with increased number of chronic diseases associated with increased psychological distress risk. Females (vs. males) and those without health insurance (vs. insured) were more likely to experience psychological distress. Immigrants (vs. non-immigrants) and racial/ethnic minorities (vs. White individuals) were less likely to experience psychological distress. There were significant interactions between chronic diseases and insurance coverage, immigration status, and race/ethnicity, but the three-way interactions were not statistically significant with psychological distress: chronic disease status vs. immigration status vs. health insurance coverage, and chronic disease vs. race/ethnicity vs. immigration status.

**Conclusion:**

The findings suggest a critical need to consider the complex ways in which chronic diseases and psychosocial factors combine to affect psychological distress and their implications for tailoring mental health screening, initiatives to reduce distress, and prevention strategies for effectively addressing health-related disparities in the general population.

## Introduction

Psychological distress, which includes anxiety and depression symptoms, the most common symptoms of mental health disorder, has been linked to high burdens of morbidity and mortality [1–9]. Psychological distress is also associated with elevated risks of negative indicators of physical health such as chronic diseases (e.g., cancer, cardiovascular diseases, stroke, atopic dermatitis, Alzheimer’s disease) and premature mortality [3, 6, 7, 10, 11]. For instance, adults with psychological distress are about two times more likely to experience mortality (age-adjusted mortality rate = 2632 deaths per 100,000 person-years) than those without psychological distress (1428 deaths) [6]. Specifically, the risks of cancer, heart disease, and Alzheimer’s disease are higher for individuals with high levels of psychological distress than those without or with lower distress [6, 12–14]. It has also contributed to higher health spending and potential years of life lost [6, 15, 16]. Mental health, including psychological distress, accounted for an estimated 5.5% (238.4 billion dollars) of the total health spending, with a 5.9% annual growth rate in 2020 in the United States (U.S.) [6, 16]. Individuals with psychological distress (vs. without psychological distress) have, on average, 3.8 years of potential life lost [6, 15]. Identifying clinical risk factors (e.g., chronic diseases) and social disparities in psychological distress is necessary for developing tailored clinical and public mental health interventions to improve overall health outcomes and quality of life, and to reduce potential adverse impacts of psychological distress on mortality rates.

The growing literature indicates that while psychological distress contributes to the burden of chronic diseases and mortality, these conditions also increase the risk of experiencing psychological distress. Individuals with chronic physical health conditions, particularly those with more symptomatic or multiple chronic conditions, have elevated risks of experiencing psychological distress [2, 15, 17–21]. A national study, for example, found that these individuals with chronic conditions are twice as likely to experience psychological distress as the general population [2]. Some of the chronic conditions linked to psychological distress include cancer, asthma, arthritis, stroke, diabetes, lung disease, heart disease, and hypertension [2, 15, 17, 19, 20]. There are biological mechanisms linking some chronic conditions to increasing levels of high blood sugar, blood pressure, and disruptions in blood circulation, that can lead to interference in psychological and emotional functioning, as well as, emotional stress and chronic pain that have also been associated with developing psychological distress [2].

Sociodemographic differences in psychological distress have been reported, with individuals residing in poor socioeconomic conditions having increased risks of psychological distress [1, 2, 22–26]. Younger, lower income and education, uninsured, racial/ethnic minority, and female individuals are more likely to experience psychological distress [1, 2, 22–25]. Studies have generally reported better health outcomes, including lower psychological distress, among immigrants compared to their native-born counterparts [11, 27–35]. However, the literature on immigration status-related disparities in psychological distress is evolving, necessitating more studies to better understand the factors contributing to the mental health of immigrants who are largely understudied. According to the healthy immigrant effect hypothesis or theory, immigrants often experience physical and behavioral health advantages compared to their native-born counterparts, although there is limited information on whether this advantage extends to mental health [28, 34, 36]. A systematic review suggests that there is an inconsistent evidence of immigrants’ mental health advantage, emphasizing the need for more studies to improve the literature [36]. Poor health behaviors such as lack of physical activity, cigarette smoking, and substance use (e.g., alcohol) are also risk factors for psychological distress [25, 26, 37, 38].

The influence of the intersection of chronic physical health conditions/diseases and sociodemographic factors on psychological distress has received little research attention. Minority identity and low socioeconomic status, especially multiple disadvantaged conditions, increase the risk of poor health outcomes, including chronic physical conditions and mental health [39–45]. Minority stress theory, for example, states that having multiple minority identities or poor socioeconomic circumstances increases the risks of health disadvantages, outcomes, and disparities [46–49]. Populations with poorer socioeconomic conditions and adversely affected by health disparities include females, racial/ethnic minority individuals, immigrants, and individuals of lower socioeconomic status (SES) [39–43, 45]. Female (vs. male) and Black (vs. White) individuals generally have higher burdens of chronic diseases and multimorbidity than their counterparts [50, 51]. For example, chronic obstructive pulmonary disease (COPD), cancer, cardiovascular disease, asthma, hypertension, arthritis, osteoporosis, diabetes, autoimmune disease, psychological distress, depression, and dementia occur more frequently in female and Black individuals [50, 51]. Specifically, the incidence rates of accumulation of chronic diseases and multiple chronic diseases are higher among females than males, especially Black females [51]. The limited evidence suggests that females with chronic conditions have higher risks of experiencing psychological distress than males with chronic conditions [2, 4, 22, 52]. Although Black individuals (vs. White individuals) are more burdened with chronic diseases and psychological distress, the intersection of multiple chronic disease and race/ethnicity on psychological distress is less understood. For instance, one national study found that Black individuals with stroke were less likely than their White counterparts to experience psychological distress [4]. High burdens of chronic physical conditions contribute significantly to high burdens of health disparities and poor health outcomes among racial/ethnic minority populations, particularly Black or African American populations [45, 53]. Insurance coverage improves access to healthcare and supports proper management and treatment of chronic diseases and psychological distress [54]. Describing and exploring the intersectionality of chronic disease status and insurance coverage, chronic disease status and sex, or chronic disease and race/ethnicity with psychological distress is essential. Additionally, while immigrants may experience better health outcomes than non-immigrants, how immigration status combines with chronic disease status or race/ethnicity to affect psychological distress is not well understood or explored. That is, immigrants have better chronic disease and mental health profiles than their native-born peers despite immigrants having more socioeconomic disadvantages such as lack of insurance and access to culturally sensitive healthcare [41, 55]. Nonetheless, the intersection of immigration status, race/ethnicity, and chronic disease status on psychological distress has received relatively little attention. Perhaps individuals with multiple minority identities, complex identities that further marginalize individuals and subject them to poorer socioeconomic conditions, as explained by the minority stress theory [42, 46–49], may elevate the risks of psychological distress.

In the present study, we examined the clinical, behavioral, and sociodemographic risk factors for psychological distress in a nationally representative sample of U.S. adults. We sought to augment the growing literature on the determinants of mental health to aid tailored clinical and public mental health treatment, reduction, and prevention strategies. Specifically, we evaluated (1) the association between multiple chronic diseases and psychological distress and (2) the ways in which chronic diseases combine with sex, race/ethnicity, immigration status, and insurance coverage to affect the risks of psychological distress. We hypothesized that; (1) multiple chronic diseases (vs. no chronic disease) will be associated with higher risks of psychological distress; (2) there will be interactions between chronic conditions and health insurance coverage, sex, race/ethnicity, and immigration status in predicting the levels of psychological distress; (3) there will be a three-way interaction of chronic diseases with insurance coverage, and with immigration status in predicting the risk of psychological distress. We hypothesized that immigrants with chronic diseases and lower insurance coverage will experience higher psychological distress than non-immigrant individuals without chronic diseases and insurance coverage; and (4) there will be a three-way interaction of chronic diseases with race/ethnicity and with immigration status in predicting the risk of psychological distress, with racial/ethnic minority immigrant individuals with chronic diseases experiencing a higher psychological distress than White non-immigrant individuals without chronic diseases.

## Methods

### Study design and sample

We analyzed the 2005–2018 National Health Interview Survey (NHIS) cross-sectional datasets, sponsored by the National Center for Health Statistics [56] at the Centers for Disease Control and Prevention [57]. The NCHS contracts the U.S. Census Bureau to conduct the survey annually among a nationally representative sample of U.S. civilian noninstitutionalized population residing within the 50 states and the District of Columbia at the time of the interview [56, 58, 59]. The survey is conducted among children aged 17 years or younger and adults aged 18 years or older. NHIS assesses public health information such as mental health, health conditions and diseases, healthcare utilization, substance use (e.g., alcohol, tobacco), and sociodemographic characteristics (e.g., age, citizenship, race/ethnicity) [56, 59]. The survey uses stratified, complex, and geographically clustered sampling techniques to select the sample of dwelling units by partitioning the U.S. into several nested levels of strata and clusters [56, 59]. First, the country is divided into counties, a small group of contiguous counties, or a metropolitan area that is located within state boundaries. Second, populous states are divided into two strata: urban and rural counties. Third, within each stratum, clusters of addresses or houses are systematically chosen from each state. Additionally, a child and an adult are randomly selected from each household. This current analysis included adults aged 18 years or older in the 2005 to 2018 surveys, with response rates as high as 69.0% in 2005 and 70.8% in 2006 to as low as 53.0% in 2017 and 53.1% in 2018. We pooled the data to increase the sample size of subpopulations (e.g., foreign-born individuals) with small samples. We performed a complete case analysis based on psychological distress (*n* = 351,457). We could not include data beyond 2018 because the content and structure of the 2019 NHIS were redesigned to improve the survey methodology and measurement (e.g., shortening the questionnaire to minimize respondent burden).

### Measures

Psychological distress status was measured with the Kessler 6-item scale. The participants were asked how often in the past 30 days they feel sad, nervous, restless, hopeless, effort, or worthless [15, 60, 61]. The self-reported options include 1 = all of the time, 2 = most of the time, 3 = some of the time, 4 = a little of the time, and 5 = none of the time. We reversed and recoded the response values (i.e., 0 = none of the time, 1 = a little of the time, 2 = some of the time, 3 = most of the time, and 4 = all of the time) to determine psychological distress scores, with a total score ranging from 0 to 24 [15, 60, 61]. Individuals with scores of *≥* 5 were considered to experience moderate to severe psychological distress; otherwise, they experienced no to low psychological distress [15, 60, 61] as we analyzed in this study.

Chronic disease status (yes/no) includes 11 diseases: asthma, arthritis, cancer, emphysema, chronic bronchitis, coronary heart disease, diabetes or sugar diabetes, hepatitis, hypertension, stroke, and weak or failing kidneys. The participants were asked to report whether a doctor or other health professional had ever told them they had any of those conditions. We created a composite score for the chronic diseases: people who had none, 1–2 diseases, and *≥* 3 diseases.

The sociodemographic factors included in the analysis are age, biological sex (male or female), race/ethnicity (Hispanic/Latino, non-Hispanic White, non-Hispanic Black, non-Hispanic Asian, or non-Hispanic other/multi-racial group), birthplace/nativity/immigration status (non-immigrant [U.S.-born] or immigrant [foreign-born]), marital status (Divorced, separated, widowed, married/living with a partner, or single/never married), U.S. census region of residence (Northeast, Midwest, West, or South), employment status (employed or unemployed), health status (insured or uninsured), education (Less than high school, high school graduate, some college/associate degree, or *≥* college degree), and poverty level (Below poverty threshold or at/above poverty threshold). We also examined body mass index (BMI [Underweight/normal, overweight, or obese]) as a health factor. Additionally, we examined health behaviors such as alcohol drinking status and cigarette smoking status. Alcohol drinking status includes non-user/lifetime abstainer (those who had fewer than 12 drinks in a lifetime), a former user (had at least 12 drinks in any one year in their lifetime but no drinks in the past year), and current user (had 1–11 or more drinks in the past year). Smoking status was determined as a never user (if the participants never smoked 100 cigarettes in their lifetime), a former user (if they ever smoked 100 cigarettes in their lifetime but currently do not smoke cigarettes), and a current user (if they ever smoked 100 cigarettes in their lifetime and currently smoke cigarettes).

### Statistical analyses

**Fig. 1:**
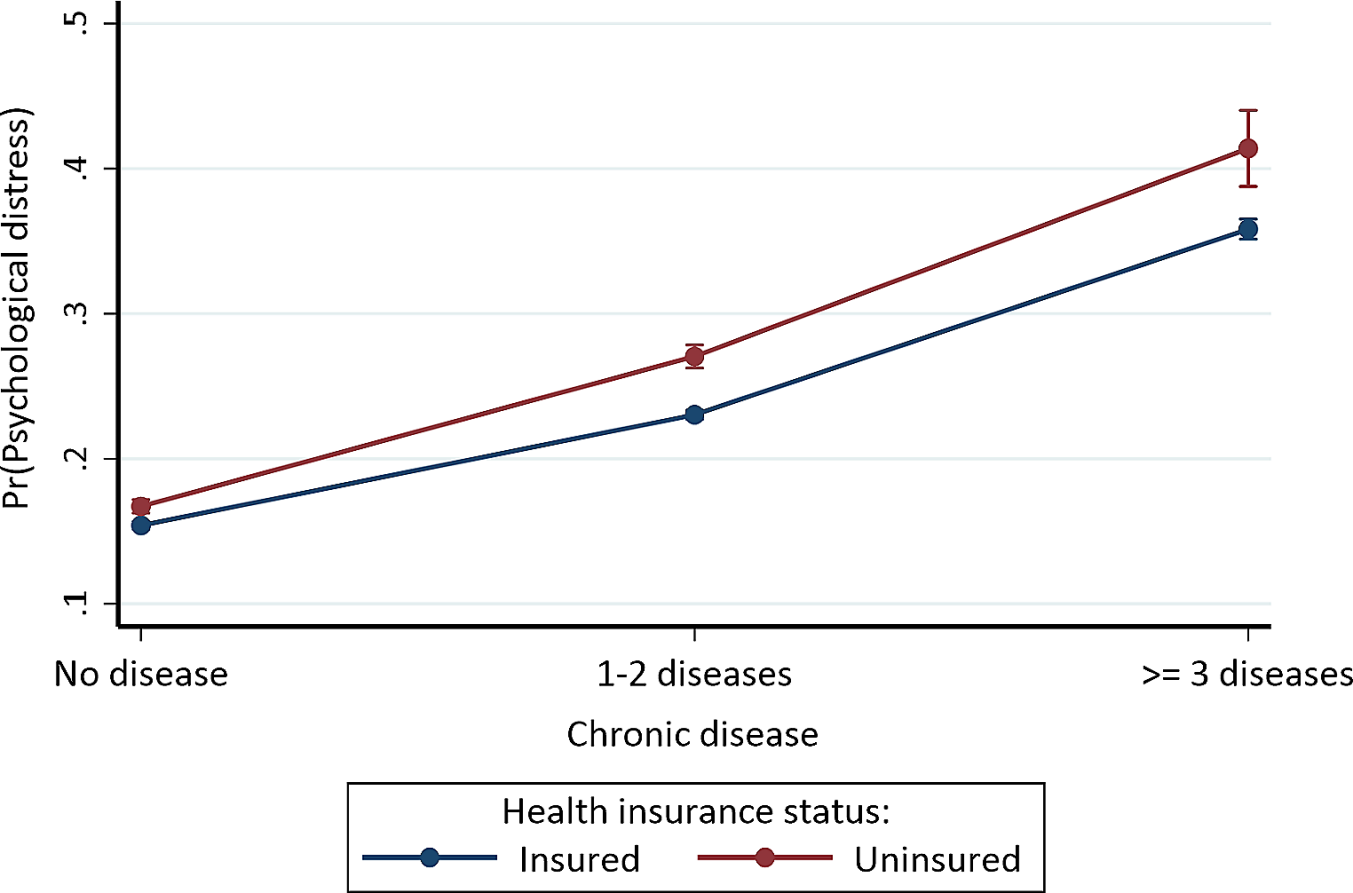
Predictive margins or average predicted probability (with 95% CIs) of psychological distress for each level of interaction of chronic disease and health insurance status

**Fig. 2:**
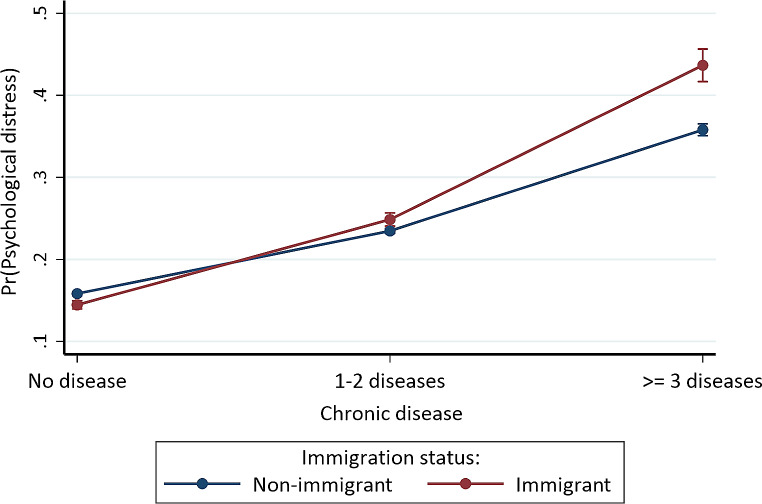
Predictive margins or average predicted probability (with 95% CIs) of psychological distress for each level of interaction of chronic disease and immigration status

**Fig. 3:**
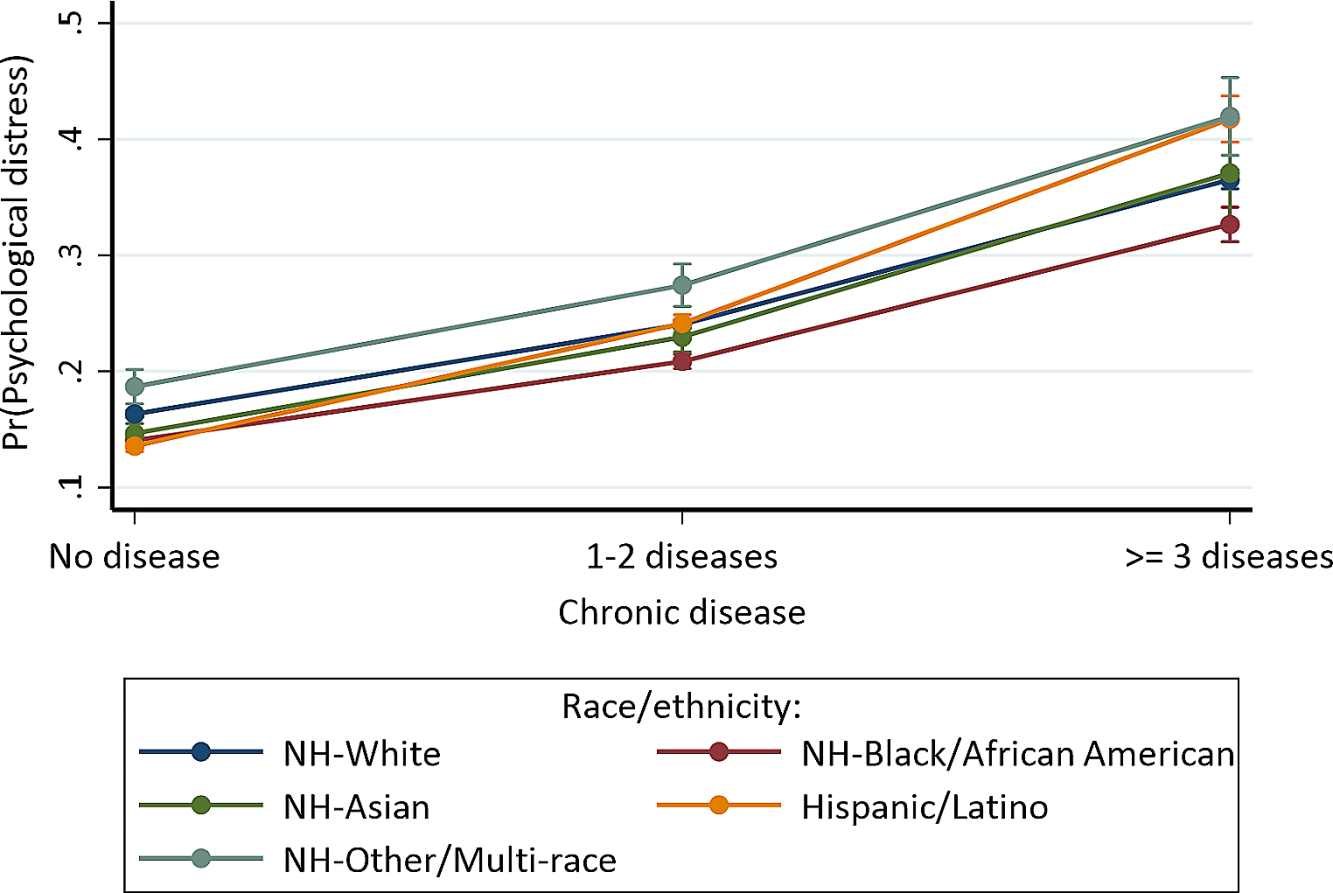
Predictive margins or average predicted probability (with 95% CIs) of psychological distress for each level of interaction of chronic disease and race/ethnicity

We used STATA version 17.0 to perform the analyses. The analytical procedures incorporated the NHIS design and adult sampling weight to obtain statistically accurate estimates and nationally representative estimations of the noninstitutionalized U.S. adult population [56]. The sampling weight for the 14 pooled survey datasets was adjusted to produce average population estimates across the 14 years and to account for the survey year effects [56]. We first calculated the descriptive statistics for the sociodemographic characteristics and health behaviors/factors (Table 1). Next, we performed bivariate statistics using chi-squared tests to determine differences in the prevalence of psychological distress by sociodemographic characteristics and health behaviors/factors (Table 1). We then fitted three sequential multivariable logistic regression models to determine the associations of chronic diseases and age with psychological distress in the model at subsequent stages (Table 2). In the first model (Model A), the association of chronic diseases and age with distress were examined. The second model (Model B) included all the sociodemographic factors in addition to the variables in Model A. The last model (Model C) entered health behaviors, Model B, and Model A. We also fitted six interaction models (Table 3) to determine the interaction between chronic diseases and health insurance status (Model D), sex (Model E), immigration status (Model F), race/ethnicity (Model G), three-way interactions of chronic diseases, health insurance status, and immigration status (Model H), and three-way interactions of chronic diseases, race/ethnicity, and immigration status (Model I). We adjusted for the Model-C factors in the interaction models. We computed the average predicted probabilities or the overall predictive margins (estimated with margins command in STATA) for the statistically significant interactions from the interaction models (i.e., Models D-I) to indicate the interaction effects. The predicted probabilities are represented in graphs with marginsplots (Figs. 1, 2 and 3). We reported unweighted frequencies and weighted percentages for the descriptive and bivariate statistics. Odds ratios (ORs) with the corresponding 95% confidence intervals (95% CIs) were estimated for the logistic regression models. The Wald test or Wald Chi-squared test was used to assess the statistical significance of the interaction terms. The level of statistical significance was determined at *p* < 0.05 for the regression models. We determined the bivariate statistical significance levels at *p* < 0.0005 to minimize the uncertainty (i.e., missed significance with small samples and higher significance with large samples) in significant estimates using large samples.

**Table 1:** Psychological distress prevalence by sociodemographic and behavioral characteristics of U.S. adults (*n* = 351,457)

	Overall sample	Psychological distress		
		No	Yes	
	*N* (%)	*n* (%)	*n* (%)	*p*-value
		278,556 (79.436)	72,901 (20.564)	
**Chronic disease**				< 0.0005
No chronic disease	176,603 (50.428)	148,388 (84.037)	28,215 (15.963)	
1–2 chronic diseases	143,647 (40.753)	110,466 (77.189)	33,181 (22.811)	
*≥* 3 chronic diseases	31,207 (8.8182)	19,702 (63.503)	11,505 (36.497)	
**Health insurance status**				< 0.0005
Uninsured	51,892 (13.478)	38,420 (73.188)	13,472 (26.812)	
Insured	299,565 (86.522)	240,136 (80.409)	59,429 (19.591)	
**Immigration status**				< 0.0005
Non-immigrant (US-born)	288,375 (84.974)	227,203 (79.067)	61,172 (20.933)	
Immigrant (Foreign-born)	63,082 (15.026)	51,353 (81.519)	11,729 (18.481)	
**Age**				< 0.0005
18–44 years old	158,603 (45.688)	124,804 (78.671)	33,799 (21.329)	
45–54 years old	60,835 (17.273)	46,961 (77.681)	13,874 (22.319)	
55–64 years old	57,449 (16.249)	44,693 (78.286)	12,756 (21.714)	
≥ 65 years old	74,570 (20.79)	62,098 (83.472)	12,472 (16.528)	
**Sex**				< 0.0005
Female	191,050 (53.251)	146,830 (77.066)	44,220 (22.934)	
Male	160,407 (46.749)	131,726 (82.134)	28,681 (17.866)	
**Race/Ethnicity**				< 0.0005
NH-White	219,808 (69.509)	174,936 (79.721)	44,872 (20.279)	
NH-Black/African American	47,806 (11.854)	37,211 (78.172)	10,595 (21.828)	
NH-Asian	18,911 (4.3068)	16,021 (84.446)	2,890 (15.554)	
Hispanic/Latino	56,801 (12.237)	44,691 (78.908)	12,110 (21.092)	
NH-Other/Multi-race	8,131 (2.093)	5,697 (69.877)	2,434 (30.123)	
**Marital status**				< 0.0005
Divorced/Separated/Widowed	90,622 (25.226)	67,378 (74.685)	23,244 (25.315)	
Married/living with a partner	179,232 (50.979)	148,909 (83.193)	30,323 (16.807)	
Single/Never married	81,603 (23.794)	62,269 (76.422)	19,334 (23.578)	
**Region of residence**				< 0.0005
Northeast	56,694 (17.023)	45,278 (80.407)	11,416 (19.593)	
North Central/Midwest	77,785 (24.124)	61,668 (79.158)	16,117 (20.842)	
South	127,646 (36.785)	101,051 (79.56)	26,595 (20.44)	
West	89,332 (22.068)	70,559 (78.782)	18,773 (21.218)	
**Employment status**				< 0.0005
Employed	212,259 (61.445)	176,559 (83.2)	35,700 (16.8)	
Not employed	139,198 (38.555)	101,997 (73.435)	37,201 (26.565)	
**Education**				< 0.0005
Less than high school	52,278 (12.938)	37,593 (71.376)	14,685 (28.624)	
High school graduate	89,568 (25.201)	69,466 (77.554)	20,102 (22.446)	
Some college/AA degree	107,368 (31.014)	83,839 (78.072)	23,529 (21.928)	
*≥*College degree	102,243 (30.847)	87,658 (85.724)	14,585 (14.276)	
**Poverty status**				< 0.0005
Below poverty threshold	56,700 (14.666)	37,209 (65.295)	19,491 (34.705)	
At or above the poverty threshold	294,757 (85.334)	241,347 (81.866)	53,410 (18.134)	
**BMI**				< 0.0005
Underweight/Normal (BMI < 25)	126,780 (36.879)	102,154 (80.705)	24,626 (19.295)	
Overweight (BMI *≥* 25 & BMI < 30)	121,756 (34.553)	99,065 (81.498)	22,691 (18.502)	
Obese (BMI 30+)	102,921 (28.568)	77,337 (75.302)	25,584 (24.698)	
**Alcohol drinking status**				< 0.0005
Never	70,844 (18.517)	57,610 (81.312)	13,234 (18.688)	
Former	53,696 (14.811)	39,638 (73.805)	14,058 (26.195)	
Current	226,917 (66.672)	181,308 (80.165)	45,609 (19.835)	
**Smoking status**				< 0.0005
Never	206,456 (58.055)	171,128 (83.111)	35,328 (16.889)	
Former	79,900 (23.179)	63,542 (79.825)	16,358 (20.175)	
Current	65,101 (18.766)	43,886 (67.585)	21,215 (32.415)	

Frequencies are unweighted and percentages are weighted

**Table 2:** Multivariable logistic regression analysis of psychological distress and its risk factors

	Model A	Model B	Model C
	OR (95% CI)	OR (95% CI)	OR (95% CI)
**Chronic disease**			
No chronic disease	Ref	Ref	Ref
1–2 chronic diseases	1.939*** (1.893, 1.986)	1.830*** (1.785, 1.875)	1.741*** (1.699, 1.785)
*≥* 3 chronic diseases	4.847*** (4.671, 5.029)	3.809*** (3.670, 3.953)	3.419*** (3.292, 3.550)
**Health insurance status**			
Uninsured		Ref	Ref
Insured		0.797*** (0.774, 0.821)	0. 851*** (0.826, 0.877)
**Immigration status**			
Non-immigrant (US-born)		Ref	Ref
Immigrant (Foreign-born)		0.913** (0.879, 0.948)	1.007 (0.969, 1.046)
**Age**			
18–44 years old	Ref	Ref	Ref
45–54 years old	0.865*** (0.842, 0.888)	0.906*** (0.881, 0.931)	0.880*** (0.856, 0.904)
55–64 years old	0.690*** (0.670, 0.712)	0.647*** (0.626, 0. 668)	0.644*** (0.623, 0.665)
≥ 65 years old	0.403*** (0.390, 0.416)	0.282*** (0.271, 0.293)	0.311*** (0.299, 0.323)
**Sex**			
Female		1.287*** (1.260, 1.314)	1.376*** (1.346, 1.406)
Male		Ref	Ref
**Race/Ethnicity**			
NH-White		Ref	Ref
NH-Black/African American		0.787*** (0.762, 0.813)	0.824*** (0.797, 0.851)
NH-Asian		0.840** (0.793, 0.890)	0.911** (0.860, 0.966)
Hispanic/Latino		0.843*** (0.811, 0.875)	0. 912*** (0.878, 0.947)
NH-Other/Multi-race		1.239*** (1.149, 1.335)	1.213*** (1.128, 1.304)
**Marital status**			
Divorced/Separated/Widowed		1.175*** (1.139, 1.212)	1.091*** (1.057, 1.126)
Married/living with a partner		0.775*** (0.753, 0.797)	0.750*** (0.729, 0.771)
Single/Never married		Ref	Ref
**Region of residence**			
Northeast		Ref	Ref
North Central/Midwest		1.005(0.965, 1.047)	0.995 (0.955, 1.035)
South		0.946** (0.911, 0.983)	0.956* (0.921, 0.992)
West		1.097*** (1.052, 1.145)	1.116*** (1.071, 1.164)
**Employment status**			
Employed		Ref	Ref
Not employed		1.777*** (1.732, 1.823)	1.778*** (1.733, 1.825)
**Education**			
Less than high school		1.730*** (1.664, 1.798)	1.530*** (1.470, 1.592)
High school graduate		1.408*** (1.366, 1.452)	1.260*** (1.223, 1.299)
Some college/AA degree		1.342*** (1.305, 1.381)	1.242*** (1.207, 1.278)
*≥*College degree		Ref	Ref
**Poverty status**			
Below poverty threshold		Ref	Ref
At or above the poverty threshold		0. 666*** (0. 647, 0.687)	0.678*** (0.658, 0.698)
**BMI**			
Underweight/Normal (BMI < 25)			Ref
Overweight (BMI *≥* 25 & BMI < 30)			1.007 (0.982, 1.032)
Obese (BMI 30+)			1.177*** (1.147, 1.209)
**Alcohol drinking status**			
Never			Ref
Former			1.377*** (1.328, 1.427)
Current			1.209*** (1.173, 1.247)
**Smoking status**			
Never			Ref
Former			1.204*** (1.170, 1.238)
Current			1.819*** (1.770, 1.868)

OR = Odds ratio. 95% CI = 95% confidence interval. Statistical significance at *p < 0.05,**p < 0.01, and ***p < 0.001

*Ref = reference*

**Model A**: Chronic disease + age

**Model B: Model A**  + sociodemographic variables (i.e., immigration status, marital status, race/ethnicity, region of residence, education, employment status, health insurance, & poverty status)

**Model C: Model A + Model B** +  health behavior variables (i.e., alcohol, BMI, & smoking status)

**Table 3:** Interaction effect of chronic diseases, insurance coverage, sex, race/ethnicity, and immigration status, adjusting for sociodemographic factors and health behaviors

Model D: Chronic disease X insurance status		Model E: Chronic disease X sex		Model F: Chronic disease X immigration status		Model G: Chronic disease X race/ethnicity	
	OR (95% C)		OR (95% C)		OR (95% C)		OR (95% C)
*Main effects*:		*Main effects*:		*Main effects*:		*Main effects*:	
**Chronic disease**		**Chronic disease**		**Chronic disease**		**Chronic disease**	
No chronic disease	Ref	No chronic disease	Ref	No chronic disease	Ref	No chronic disease	Ref
1–2 chronic diseases	1.933*** (1.831, 2.040)	1–2 chronic diseases	1.762*** (1.699, 1.828)	1–2 chronic diseases	1.691*** (1.647, 1.736)	1–2 chronic diseases	1.684*** (1.635, 1.734)
*≥* 3 chronic diseases	3.895*** (3.453, 4.394)	*≥* 3 chronic diseases	3.304*** (3.124, 3.495)	*≥* 3 chronic diseases	3.242*** (3.116, 3.372)	*≥* 3 chronic diseases	3.228*** (3.090, 3.371)
**Health insurance status**		**Sex**		**Immigration status**		**Race/ethnicity**	
Uninsured	Ref	Male	Ref	Non-immigrant	Ref	NH-White	Ref
Insured	0.902*** (0.868, 0.937)	Female	1.379*** (1.334, 1.426)	Immigrant	0.893*** (0.853, 0.934)	NH-Black/African American	0.828*** (0.787, 0.871)
*Interaction effects*:	F (2, 1244) = 10.76, *p* < 0.001	*Interaction effects*:	F (2, 1244) = 2.77, *p* = 0.063	*Interaction effects*:	F (2, 1244) = 57.11, *p* < 0.001	NH-Asian	0.872*** (0.810, 0.940)
No chronic disease X uninsured	Ref	No chronic disease X male	Ref	No chronic disease X non-immigrant	Ref	Hispanic/Latino	0.793*** (0.756, 0.833)
1–2 chronic diseases X insured	0.880*** (0.830, 0.932)	1–2 chronic diseases X female	0.979 (0.937, 1.023)	1–2 chronic diseases X immigrant	1.216*** (1.151, 1.285)	NH-Other/Multi-race	1.191** (1.074, 1.321)
*≥* 3 chronic diseases X insured	0.858* (0.758, 0.972)	*≥* 3 chronic diseases X female	1.059 (0.989, 1.135)	*≥* 3 chronic diseases X immigrant	1.606*** (1.457, 1.771)	*Interaction effects*:	F (8, 1238) = 15.18, *p* < 0.001
						No chronic disease X NH-White	Ref
						1–2 chronic diseases X NH-Black/African American	0.990 (0.930, 1.054)
						1–2 chronic diseases X NH-Asian	1.073 (0.969, 1.189)
						1–2 chronic diseases X Hispanic/Latino	1.266*** (1.193, 1.344)
						1–2 chronic diseases X NH-Other/Multi-race	1.017 (0.894, 1.157)
						*≥* 3 chronic diseases X NH-Black/African American	1.002 (0.916, 1.096)
						*≥* 3 chronic diseases X NH-Asian	1.177 (0.945, 1.465)
						*≥* 3 chronic diseases X Hispanic/Latino	1.603*** (1.448, 1.774)
						*≥* 3 chronic diseases X NH-Other/Multi-race	1.079 (0.901, 1.292)
**Model H: Chronic disease X health insurance status X immigration status**			**Model I: Chronic disease X race/ethnicity X immigration status**				
*Main effects*:			*Main effects*:				
**Chronic disease**			**Chronic disease**				
No chronic disease	Ref		No chronic disease	Ref			
1–2 chronic diseases	1.852*** (1.741, 1.971)		1–2 chronic diseases	1.676*** (1.626, 1.727)			
*≥* 3 chronic diseases	3.552*** (3.117, 4.046)		*≥* 3 chronic diseases	3.193*** (3.053, 3.339)			
**Health insurance status**			**Race/ethnicity**				
Uninsured	Ref		NH-White	Ref			
Insured	0.831*** (0.795, 0.868)		NH-Black/African American	0.826*** (0.783, 0.872)			
**Immigration status**			NH-Asian	0.900 (0.789, 1.027)			
Non-immigrant	Ref		Hispanic/Latino	0.909** (0.854, 0.967)			
immigrant	0.737*** (0.683, 0.795)		NH-Other/Multi-race	1.184** (1.061, 1.320)			
*Interaction effects*:							
**Chronic disease X health insurance status**			**Immigration status**				
No chronic disease X uninsured	Ref		Non-immigrant	Ref			
1–2 chronic diseases X insured	0.901** (0.843, 0.963)		immigrant	1.037 (0.949, 1.133)			
*≥* 3 chronic diseases X insured	0.907 (0.793, 1.038)		*Interaction effects*:				
			**Chronic disease X race/ethnicity**				
**Chronic disease X immigration status**			No chronic disease X NH-White	Ref			
No chronic disease X non-immigrant	Ref		1–2 chronic diseases X NH-Black/African American	0.995 (0.931, 1.063)			
1–2 chronic diseases X immigrant	1.071 (0.954, 1.203)						
*≥* 3 chronic diseases X immigrant	1.422* (1.015, 1.992)		1–2 chronic diseases X NH-Asian	0.991 (0.810, 1.214)			
**Health insurance X immigration status**			1–2 chronic diseases X Hispanic/Latino	1.150** (1.054, 1.255)			
Uninsured X non-immigrant	Ref		1–2 chronic diseases X NH-Other/Multi-race	1.023 (0.896, 1.168)			
Insured X immigrant	1.329*** (1.221, 1.446)		*≥* 3 chronic diseases X NH-Black/African American	1.007 (0.917, 1.105)			
**Chronic disease X health insurance X immigration status**	F (2, 1244) = 1.03, *p* = 0.356		*≥* 3 chronic diseases X NH-Asian	0.944 (0.625, 1.427)			
No chronic disease X uninsured X non-immigrant	Ref		*≥* 3 chronic diseases X Hispanic/Latino	1.253** (1.081, 1.453)			
1–2 chronic diseases X insured X immigrant	1.103 (0.965, 1.260)		*≥* 3 chronic diseases X NH-Other/Multi-race	1.070 (0.892, 1.283)			
*≥* 3 chronic diseases X insured X immigrant	1.047 (0.740, 1.482)						
			**Chronic disease X Immigration status**				
			No chronic disease X non-immigrant	Ref			
			1–2 chronic diseases X immigrant	1.114 (0.980, 1.268)			
			*≥* 3 chronic diseases X immigrant	1.351** (1.117, 1.634)			
			**Race/ethnicity X immigration status**				
			NH-White X immigrant	Ref			
			NH-Black/African American X immigrant	0.992 (0.840, 1.171)			
			NH-Asian X immigrant	0.937 (0.791, 1.109)			
			Hispanic/Latino X immigrant	0.762*** (0.675, 0.859)			
			NH-Other/Multi-race X immigrant	1.043 (0.746, 1.457)			
			**Chronic disease X race/ethnicity X immigration status**	F (8, 1238) = 0.59, *p* = 0.786			
			No chronic disease X NH-White X immigrant	Ref			
			1–2 chronic diseases X NH-Black/African American	0.903 (0.705, 1.157)			
			1–2 chronic diseases X NH-Asian X immigrant	1.006 (0.773, 1.308)			
			1–2 chronic diseases X Hispanic/Latino X immigrant	1.068 (0.903, 1.264)			
			1–2 chronic diseases X NH-Other/Multi-race X immigrant	0.912 (0.571, 1.456)			
			*≥* 3 chronic diseases X NH-Black/African American X immigrant	0.864 (0.567, 1.314)			
			*≥* 3 chronic diseases X NH-Asian X immigrant	1.011 (0.603, 1.698)			
			*≥* 3 chronic diseases X Hispanic/Latino X immigrant	1.182 (0.899, 1.552)			
			*≥* 3 chronic diseases X NH-Other/Multi-race X immigrant	1.380 (0.555, 3.429)			

OR = Odds ratio. 95% CI = 95% confidence interval. Statistical significance at *p < 0.05, **p < 0.01, and *** *p* < 0.001

*Ref = reference*

**Mode D (Interaction Term)**: Chronic disease x insurance status

**Model E (Interaction Term)**: Chronic disease x sex

**Mode F (Interaction Term)**: Chronic disease x immigration status

**Model G (Interaction Term)**: Chronic disease x race/ethnicity

**Model H (Interaction Term)**: Chronic disease x insurance status x immigration status

**Model I (Interaction Term)**: Chronic disease x race/ethnicity x immigration status

## Results

### Population characteristics

Table 1 shows the distribution of the sociodemographic characteristics and health factors/behaviors and the prevalence of psychological distress among the U.S. adult population. Most of the population were U.S.-born individuals (84.974%), aged 18–44 years (45.688%), females (53.251%), non-Hispanic White persons (69.509%), married or living with a partner (50.979%), resided in the U.S. South (36.785%), employed (61.445%), had technical or some college education (31.014%), insured (86.522%), and at or above the poverty threshold (85.334%). Two-thirds currently used alcohol (66.672%). More than a third of them were overweight (34.553%) and had one to two chronic diseases (40.753%). At least 19% of them were former (23.179%) and current (18.766%) cigarette users. One-fifth of the population reported experiencing psychological distress (20.564%).

### Differences in the prevalence of psychological distress

Overall, most of those who reported experiencing high levels of psychological distress were 45–54 years old, females, non-Hispanic other/multi-racial groups, divorced/separated/widowed, resided in the U.S. West, were unemployed, below the poverty threshold, had less than high school education, uninsured, and born in the U.S. (Table 1). They also were obese, former alcohol users, current cigarette smokers, and had three or more chronic diseases.

### Factors associated with psychological distress

We observed a dose-response relationship between chronic diseases and psychological distress, as the odds ratio estimates for psychological distress increased with an increased number of chronic diseases (Table 2), confirming our hypothesis. After adjusting for only age, increasing number of multiple chronic conditions was associated with higher odds of psychological distress (Model A). Similarly, the increasing number of multiple chronic conditions were associated with higher odds of psychological even after adjusting for age and other sociodemographic factors (Model B). These similar patterns of association obtained in models A and B were also observed in model C, after adjusting for age, other sociodemographic factors, and health behaviors. We interpreted the odds ratio estimates in model C, which is the full or final model.

Having 1–2 (OR = 1.741, 95% CI = 1.699, 1.785) or *≥* 3 chronic diseases (OR = 3.419, 95% CI = 3.292, 3.540) was significantly associated with experiencing psychological distress (Model C). Individuals aged 45–54 (OR = 0.880, 95% CI = 0.856, 0.904), 55–64 (OR = 0.644, 95% CI = 0.623, 0.665), and ≥ 65 (OR = 0.311, 95% CI = 0.299, 0.323) years had a lower likelihood of psychological distress compared to those aged 18–44 years. Females (versus males) had higher odds of psychological distress (OR = 1.376, 95% CI = 1.346, 1.406). Compared to non-Hispanic White individuals, non-Hispanic Black/African American (OR = 0.824, 95% CI = 0.797, 0.851), non-Hispanic Asian (OR = 0.911, 95% CI = 0.860, 0.966), and Hispanic/Latino (OR = 0.912, 95% CI = 0.878, 0.947) individuals had lower odds while non-Hispanic other/multi-racial groups (OR = 1.213, 95% CI = 1.128, 1.304) had higher odds of psychological distress. The odds were higher for those who experienced divorce/separation/widowhood (OR = 1.091, 95% CI = 1.057, 1.126) but lower for those who were married/living with a partner (OR = 0.750, 95% CI = 0.729, 0.771) compared to those who were single/never married. Individuals living in the South had a lower likelihood (OR = 0.956, 95%CI = 0.921, 0.992), while those living in the West had a higher likelihood (OR = 1.116, 95% CI = 1.071, 1.164) of experiencing psychological distress than those living in the Northeast. Being unemployed (versus employed) was associated with higher odds of experiencing psychological distress (OR = 1.778, 95% CI = 1.733, 1.825).

There was also a dose-response relationship between the level of education and psychological distress, with higher odds for having less than high school (OR = 1.530, 95% CI = 1.470, 1.592), high school graduate (OR = 1.260, 95% CI = 1.223, 1.299), and some college/AA degree (OR = 1.242, 95% CI = 1.207, 1.278) education compared to at least college degree education. Being at or above the poverty threshold (versus below the poverty threshold) was associated with lower odds of psychological distress (OR = 0.678, 95% CI = 0.658, 0.698). The lower odds were also observed among insured individuals (OR = 0.851, 95% CI = 0.826, 0.877) compared to uninsured individuals. Higher BMI, alcohol use, or cigarette smoking were associated with a higher likelihood of experiencing psychological distress.

### Intersection of chronic diseases, health insurance status, sex, immigration status, and race/ethnicity

We found significant interactions between chronic disease status and health insurance status (*p* < 0.001), immigration status (*p* < 0.001), and race/ethnicity (*p* < 0.001), respectively, which confirmed our hypothesis (Table 3). However, the interaction between chronic disease status and sex (Model E: *p* = 0.063) was not statistically significant. The three-way interactions were also not statistically significant: chronic disease with insurance status and immigration status (Model H: *p* = 0.354) and chronic disease with race/ethnicity and immigration status (Model 1: *p* = 0.786). For the interaction between chronic disease and health insurance status (Model D), having 1–2 (OR = 0.880, 95% CI = 0.830, 0.932) or *≥* 3 (OR = 0.858, 95% CI = 0.758, 0.972) chronic diseases and being insured was associated with lower odds of psychological distress compared to no chronic disease and being uninsured. Figure 1 shows the average predicted probabilities of psychological distress for each level of interaction of chronic disease and health insurance status. In general, individuals with *≥* 3 chronic diseases and who were uninsured (41.397%, *p* < 0.001) had the highest probability of psychological distress, while those with no chronic disease but insured had the lowest probability (15.403%, *p* < 0.001).

For chronic diseases with immigration status, the odds were higher for foreign-born individuals with 1–2 chronic diseases (OR = 1.216, 95% CI = 1.151, 1.285) or *≥* 3 chronic diseases (OR = 1.606, 95% CI = 1.457, 1.771) compared to US-born individuals without chronic disease (Model F). Figure 2 shows that foreign-born individuals with *≥* 3 chronic diseases (43.664%, *p* < 0.001) had the highest probability of psychological distress, while foreign-born individuals without chronic disease (14.459%, *p* < 0.001) had the lowest probability, followed by US-born persons without chronic disease (15.827%, *p* < 0.001) or with 1–2 chronic diseases (23.474%, *p* < 0.001), foreign-born individuals with 1–2 chronic diseases (24.864%, *p* < 0.001), and US-born persons with *≥* 3 chronic diseases (35.805%, *p* < 0.001).

For the interaction between chronic disease status and race/ethnicity (Model G), Hispanic/Latino individuals with 1–2 (OR = 1.266, 95% CI = 1.193, 1.344) or *≥* 3 (OR = 1.603, 95% CI = 1.448, 1.774) chronic diseases had higher odds of psychological distress compared to non-Hispanic White individuals without chronic disease. Figure 3 indicates that non-Hispanic other/multi-racial groups (41.968%, *p* < 0.001) and Hispanic/Latino (41.744%, *p* < 0.001) with *≥* 3 chronic diseases similarly had the highest probability of psychological distress. Hispanic/Latino individuals without chronic disease (13.564%, *p* < 0.001) had the lowest probability, followed by (1) non-Hispanic Black/African Americans (14.041%, *p* < 0.001), non-Hispanic Asians (14.648%, *p* < 0.001), non-Hispanic Whites (16.323%, *p* < 0.001), and non-Hispanic other/multi-racial persons (18.677%, *p* < 0.001) without chronic disease, (2) non-Hispanic Black/African Americans (20.852%, *p* < 0.001), non-Hispanic Asians (22.958%, *p* < 0.001), non-Hispanic Whites (24.051%, *p* < 0.001), Hispanic/Latinos (24.127%, *p* < 0.001), and non-Hispanic other/multi-racial individuals (27.421%, *p* < 0.001) with 1–2 chronic diseases, and (3) non-Hispanic Black/African American (32.659%, *p* < 0.001), non-Hispanic White (36.524%, *p* < 0.001), and non-Hispanic Asian (37.082%, *p* < 0.001) individuals with *≥* 3 chronic diseases.

## Discussion

In this nationally representative study of the U.S. adult population, about 1 in 5 adults experienced psychological distress, an indication of clinical need, similar to the ratio reported by CDC and other researchers [57, 62]. The elevated prevalence varied across all sociodemographic groups, especially among those with lower socioeconomic status (SES). Multiple factors, ranging from clinical, behavioral, and sociodemographic factors examined in this study, were associated with the risks of experiencing psychological distress. Specifically, multiple chronic diseases, poor health behaviors, and lower SES were associated with increased risks of psychological distress. We also found that the association between multiple chronic diseases and psychological distress was heightened among minority groups, including among uninsured and immigrant individuals, and socioeconomically disadvantaged groups like female individuals. These findings suggest the need to consider the potential impact of these risk factors, particularly as they combine with each other, in treatment, symptom reduction, and prevention strategies for psychological distress in addressing health-related disparities in the general population.

Consistent with the literature, our findings showed that lower SES was associated with higher psychological distress risks, with increased risks among younger, female, unemployed individuals, or those below the poverty threshold [22, 63]. These findings reflect the minority stress theory which predicts that poor socioeconomic circumstances, especially multiple minority deleterious conditions, are significant stressors that can negatively impact mental health outcomes, including psychological distress [46–49]. As a result, considering these stressors and intervening on them in mental health research, treatment, and prevention can enhance mental health and help reduce mental health disparities. Our findings also indicate that insured individuals, those with higher education, or married/living with a partner were less likely to experience psychological distress, implying that insurance coverage, education, and social support can be protective factors for mental well-being [15, 62]. Prior research also indicates that insured individuals without a usual source of care and uninsured individuals with psychological distress are less likely to receive mental health treatment than their insured counterparts [62]. More research is needed to better understand the variations in psychological distress by census region and to delineate the specific stress-related risk factors in geographic contexts that contribute to the observed variation by region. We found that individuals in the Southern and Midwestern census regions had lower risks, while those in the Western region had higher risks compared to their counterparts in the Northeastern region. These regional differences might be due, at least in part, to regional disparities in mental health access and treatment, with more adults receiving mental health treatment in rural or nonmetropolitan areas than in urban or large metropolitan areas [64].

As reported in previous studies, we found that racial/ethnic minority individuals had lower prevalence and risks of psychological distress than White individuals, while other/multi-racial groups had higher risks [65–69]. It is possible that while racial/ethnic minority individuals are less likely to experience mental health symptoms, including psychological distress, they may have higher burdens of mental health disorders due to a lack of access to early mental health treatment among minority individuals [46–49, 70, 71]. Prior research indicates that compared to White individuals, racial/ethnic minority individuals tend to have more serious mental illness but lower mental healthcare utilization, higher treatment attrition rates, and lower treatment improvement due partly to medical mistrust, lack of resources, and discrimination in the quantity and quality of care received [70, 71]. The higher risks of psychological distress among other/multi-racial groups may be attributable to discrimination and ethno-racial identity that elevate the risk of physical, behavioral, and mental health outcomes [72]. In addition to the social and economic challenges linked to lower educational attainment and socioeconomic status, other/multi-racial groups, American Indian/Alaskan Native or Native American, and Native Hawaiian/Pacific Islander other than Black, Asian, and Hispanic individuals, experience significant burdens of discrimination, rejection, and racial slurs [72]. These burdens can lead to low self-esteem, hopelessness, stress or distress, behavioral health problems (e.g., substance use), mental health problems, suicidal behaviors, and chronic health conditions [72]. More research, however, is needed to evaluate multi-racial subgroup differences in psychological distress, as there are different racial combinations that could inform personalized mental health interventions.

Prior research has found that poor health behaviors significantly impact health outcomes, including the risks of mental health outcomes [9, 15, 37]. In this study, we found that health-compromising behaviors such as cigarette smoking, alcohol use, and higher body mass index were also significant contributors to psychological distress. Smoking and alcohol use, for example, can lead to psychological distress due to alterations to the hypothalamic–pituitary–adrenal (HPA) axis, an interaction between the hypothalamus, pituitary, and adrenal glands responsible for adjusting the body’s homeostatic systems (e.g., metabolic, cardiovascular, immune, reproductive, and central nervous systems) in responding to stress, that can lead to psychological and physiological vulnerability to stressors [73–76]. Evidence also suggests that the disruptions in the functioning of the HPA can result in elevated levels of circulating cortisol, increasing the risk of developing obesity [74, 77, 78]. Interventions targeting these risky health behaviors may reduce the risk of developing psychological distress and other physiological vulnerabilities. However, it should be noted that mental health conditions, including psychological distress, can also contribute to increased substance use behaviors as a coping strategy to relieve stress or distress [79, 80]. Thus, mental health conditions and substance use/misuse are often comorbid or co-occurring conditions [79–81]. Integrated psychosocial interventions may be needed to treat both mental health and substance misuse behaviors to reduce and prevent relapse in treatment outcomes [81, 82].

Similar to the evolving literature, we found a dose-response relationship between chronic diseases and psychological distress, with increasing numbers of chronic diseases associated with higher risks of psychological distress [2, 15, 17–21, 62]. Our findings also contributed to our understanding that the dose-response relationship between the number of chronic diseases and psychological distress is both nuanced and multifaceted due to the range of psychosocial factors that influenced the risks of experiencing psychological distress. For instance, the risk of experiencing psychological distress decreased with the increased number of chronic diseases for insured (vs. uninsured) individuals. That is, uninsured individuals with or without chronic diseases had a higher average predicted probability of experiencing psychological distress than insured individuals with or without chronic diseases. However, within-group differences emerged; uninsured and insured persons with chronic diseases, especially those with ≥ 3 chronic diseases, had a higher probability of experiencing psychological distress than their counterparts without chronic diseases. Although sex was associated with psychological distress, sex did not significantly moderate the association between chronic diseases and psychological distress, suggesting that males and females may have similar risks of psychological distress due to the influence of chronic diseases. The odds ratios, however, suggest that considering males and females as a monolithic population may be problematic because while females with 1–2 chronic diseases (vs. males without chronic diseases) were less likely to experience psychological distress, those with *≥* 3 chronic diseases had higher risks of psychological distress than their male counterparts.

Our findings further revealed evidence of a buffering effect: the psychological distress risks increased with the increased number of chronic diseases among immigrant/foreign-born (vs. non-immigrant) individuals, but these immigrants had lower psychological distress risks when their chronic disease status was not considered with their immigration status. Specifically, we observed between- and within-group differences in psychological distress, with immigrant persons with or without chronic diseases having a higher average predicted probability of experiencing psychological distress than non-immigrant persons with or without chronic diseases. The immigrant and non-immigrant persons with chronic diseases, particularly those with at least three chronic diseases, were more likely to experience psychological distress than their counterparts without chronic diseases. The evidence that immigrants have better health outcomes than non-immigrants may not apply in every situation, thus the intersection of multiple clinical and psychosocial factors may influence the risk levels and direction of health outcomes between immigrants and non-immigrants [36]. As evident in our three-way interaction results, (1) the intersection of chronic disease status, immigration status, and health insurance coverage and (2) the intersection of chronic disease status, race/ethnicity, and immigration status had no significant effect on psychological distress, indicating no significant group differences. Further studies are needed to understand better the intersectionality of multiple risk factors on psychological distress among immigrants and non-immigrants while considering their within-group differences due to the distinct cultural characteristics, health behavior, and health perception of immigrants. Besides, research concerning immigrant mental health and their related disparities is notably lacking despite the rapidly increasing immigrant population, especially African immigrants, in the U.S.

Studies generally reported that racial/ethnic minority individuals, particularly Black/African American persons, have higher burdens of health outcomes, including chronic diseases and mental health [39–45]. These increased burdens and disparities are associated with multiple minority identities and lower SES, circumstances that predispose disadvantaged individuals to more discrimination, violence, and limited health resources and opportunities [46–49]. However, there is less information on the intersection effects of chronic diseases and race/ethnicity on psychological distress. We found significant interaction between chronic disease status and race/ethnicity, with significant between- and within-group differences in psychological distress. In general, racial/ethnic individuals with chronic diseases, particularly those with *≥* 3 chronic diseases, had higher probability of experiencing psychological distress than their peers without chronic diseases. Specifically, non-Hispanic other/multi-racial groups and Hispanic/Latino with *≥* 3 chronic diseases similarly had the highest probability of experiencing psychological distress. Hispanic/Latino persons without chronic disease had the lowest probability, followed by non-Hispanic Black/African American, non-Hispanic Asian, non-Hispanic White, and non-Hispanic other/multi-racial individuals without chronic disease. On average, non-Hispanic other/multi-racial groups with at least one chronic disease had the highest probability of psychological distress, followed by Hispanic, non-Hispanic White and Asian (these two groups had similar probability), and non-Hispanic Black individuals. These findings demonstrate the multiplicative and complex effects of chronic physical health outcomes and race/ethnicity on mental health that need to be recognized in further studies and tailored mental health interventions. More studies are also needed to evaluate the intersection between race/ethnicity and various types of common chronic physical health outcomes (e.g., cancer, stroke, cardiovascular disease, asthma, arthritis, diabetes) to identify how each chronic disease affects mental health of diverse racial/ethnic groups. The findings emphasize the importance of exploring and evaluating intersections between social identities and health outcomes in addressing heterogeneity and disparities in mental health [46–49, 83]. A study, for example, conducted quantitative analytic intersectionality of race and sexual and gender identity (SGM) in psychological distress; Black non-SGM minority (vs. White non-SGM minority) had a lower psychological distress when discrimination was not considered, but both Black SGM and non-SGM individuals (vs. White non-SGM minority) had a higher psychological distress when discrimination was considered.

The evaluation and interpretation of the findings should be considered with the following limitations in mind. We could not establish causality in this study with our use of cross-sectional data, which involved assessing both exposure and outcome variables at the same time, such that the temporal sequence could not be established. Also, the cross-sectional data were based on self-reported responses, which are potentially vulnerable to reporting bias. Third, the mental health measure, psychological distress, captures broad symptoms and, therefore, we lacked both the criteria for assessing specific psychiatric disorders, as well as the absence of an independent clinical evaluation. Fourth, residual confounders such as other mental health disorders (e.g., bipolar, schizophrenia, post-traumatic stress disorder) and sexual and gender identity, significant risk factors for psychological distress [49, 62, 84], were not examined in this study. Additionally, the findings only reflect the psychological distress among the noninstitutionalized population because institutionalized individuals were not included in the NHIS. Further studies may fill these gaps to augment the literature in addressing mental health challenges, especially among potentially vulnerable population subgroups.

## Conclusions

Our findings add to the growing literature on psychological distress and chronic physical diseases using nationally representative population data. The findings revealed a positive relationship between chronic diseases and psychological distress, with a dose-response relationship. Treating and reducing chronic diseases has the potential to significantly improve psychological distress and, ultimately, mental well-being. The significant intersection of chronic diseases, insurance coverage, immigration status, and race/ethnicity with psychological distress demonstrates the need to consider the multiplicative and complex effects of clinical and psychosocial factors for tailored mental health screening and interventions. Moreover, the significant association of behavioral risk factors such as smoking and alcohol use with distress underscores the need to promote positive health behaviors while providing personalized mental health messages and clinical treatment. Additionally, the evidence of the buffering effect observed in this study that the intersection of chronic disease and immigration status showed heightened risks among immigrant (vs. non-immigrant) individuals but had lower risks if chronic diseases were not considered requires more research to delineate the immigration status-related disparities in psychological distress.

## Data Availability

The datasets generated by the survey research during and/or analyzed during the current study are publicly available in the CDC database repository, https://www.cdc.gov/nchs/nhis/data-questionnaires-documentation.htm.
